# Chronic, Reusable, Multiday Neuropixels Recordings during Free-Moving Operant Behavior

**DOI:** 10.1523/ENEURO.0245-23.2023

**Published:** 2024-01-10

**Authors:** Zhimin Song, Abigail Alpers, Kasey Warner, Francesca Iacobucci, Eric Hoskins, John F. Disterhoft, Joel L. Voss, Alik S. Widge

**Affiliations:** ^1^ Department of Psychiatry, University of Minnesota, Minneapolis, 55455 Minnesota; ^2^ Department of Neuroscience, Northwestern University, Evanston, 60208 Illinois; ^3^ Department of Neurology, University of Chicago, Chicago, 60637 Illinois

**Keywords:** cognitive flexibility, deep brain stimulation, paired associate learning, set shift

## Abstract

Electrophysiological recording is a powerful technique to examine neuronal substrates underlying cognition and behavior. Neuropixels probes provide a unique capacity to capture neuronal activity across many brain areas with high spatiotemporal resolution. Neuropixels are also expensive and optimized for acute, head-fixed use, both of which limit the types of behaviors and manipulations that can be studied. Recent advances have addressed the cost issue by showing chronic implant, explant, and reuse of Neuropixels probes, but the methods were not optimized for use in free-moving behavior. There were specific needs for improvement in cabling/connection stability. Here, we extend that work to demonstrate chronic Neuropixels recording, explant, and reuse in a rat model during fully free-moving operant behavior. Similar to prior approaches, we house the probe and headstage within a 3D-printed housing that avoids direct fixation of the probe to the skull, enabling eventual explant. We demonstrate innovations to allow chronic headstage connection with protection against environmental factors and a more stable cabling setup to reduce the tension that can interrupt recording. We demonstrate this approach with rats performing two different behavioral tasks, in each case showing: (1) chronic single- or dual-probe recordings in free-moving rats in operant chambers and (2) reusability of Neuropixels 1.0 probes with continued good single-unit yield after retrieval and reimplant. We thus demonstrate the potential for Neuropixels recordings in a wider range of species and preparations.

## Significance Statement

Electrophysiological recordings with Neuropixels probes provide a unique capacity in system neuroscience to capture neuronal activity with high spatiotemporal resolution. Yet Neuropixels probes are quite difficult to use as they are expensive, fragile, and tricky to use, particularly in the context of chronic implants in free-moving animals. This work shows a design for a chronic probe housing that enables reuse after implantation in free-moving rats, with verification that it works across multiple uses of the same probe. We developed a protocol that specifically deals with issues relating to cabling and recording sustainability that came up in most reported approaches. This method should facilitate the usage of chronic Neuropixel recordings in future research and be of interest to the neuroscience community.

## Introduction

Extracellular electrophysiological recording is an essential tool in neuroscience, as it allows researchers to examine direct neuronal activity at the cellular level with a high temporal resolution. Silicon recording probes have been an important part of this technique since their first emergence (Drake et al., 1988) because of their capacity to carry and record from a large number of contacts. With improved techniques to fabricate silicon probes, the channel count of a recording probe has increased dramatically while maintaining a reasonable device size. The development of high-density Neuropixels 1.0 probes, with nearly 1,000 contacts per probe, enables simultaneous recording of hundreds of individual neurons over a distance of 10 mm with high spatiotemporal resolution (Jun et al., 2017).

Although Neuropixels probes were initially used in both acute head-fixed and free-moving animal recordings, their predominant use in the literature is in head-fixed preparations. These preparations are more easily compatible with very delicate silicon shanks. Recordings in unrestrained animals, collected over days to months, are however a common neuroscience technique. These free-moving recordings provide opportunities to investigate associations between neural markers and behavioral or cognitive traits, without concerns that the behavior may be disrupted by the unnatural situation of head fixation. Probe implantation for these chronic recordings requires that the implant securing system (e.g., any holding device or adhesive headcap) is both durable and small enough to fit on the head of the animal. The latter size constraint can become limiting when multiple probes will be used on a single animal, particularly in the small rodents most commonly used in neuroscience. Further, for Neuropixels specifically, the cost per probe is very high, and most laboratories would be unable to afford an experiment where each probe was used only once. However, multiple groups have shown Neuropixels reuse after chronic implant, by developing a setup that can temporarily fix the probe to the skull during the chronic implant and then later allow explantation after recordings are completed (Juavinett et al., 2019; Luo et al., 2020; van Daal et al., 2021). All of these designs center around the concept of a 3D-printed plastic housing that protects the Neuropixels and allows them to be withdrawn from the brain without interference from surrounding acrylic.

The next major need was adapting these approaches to multi-probe recordings. Systems neuroscience is increasingly concerned with communication and interaction between multiple brain regions (Buzsáki, 2004; Blanche et al., 2005; Hultman et al., 2018), which can best be measured with probe implantation along multiple vertical trajectories. It is infeasible to place separate housings for these trajectories, as their bulk would not fit on a rat or mouse skull. Some existing Neuropixels housing designs for chronic recordings are capable of holding multiple probes while allowing explantation and reuse (Juavinett et al., 2019; Luo et al., 2020; van Daal et al., 2021). However, these designs still have challenges. These include a weak connection between the serial transmission cable and headstage, leading to frequent disconnections when a free-moving animal places tension on that cable. Some of the existing designs also require re-connecting the probe to the headstage each day (Luo et al., 2020). This is challenging, as it requires precise manipulation of a very small part while the animals are moving. In our experience, a reused probe could require several clampings to be detected by the recording software. Further, repetitive clamping with the zero-insertion-force (ZIF) connector in the Neuropixels headstage often leads to damaged connections due to repeated mechanical actuation of a small, fragile part.

We now overcome those limitations with a new design optimized for free-moving rat studies where rats perform complex operant tasks. As with the previously reported housing designs, our design is a 3D-printed housing that enables explantation after usage. The new design also allows close to zero tension on the cable and the Omnetics connector, ensuring uninterrupted recordings. Because not all Neuropixels-compatible recording systems contain commutators, we developed an intermediate solution to prevent cable tangling and to keep cables out of animals’ reach. Additionally, our design keeps the headstage chronically connected with the probe to avoid repetitive clamping, while resolving the problem of maintaining delicate electronics in direct contact with a humid craniotomy/chamber environment. We demonstrate this system in two preparations: hippocampal recording during a touch screen spatial learning task and dual cortical/striatal recording during an extradimensional set-shifting task. We specifically show recovery of behaviorally relevant signals before and after probe explant and reuse.

## Materials and Methods

### Animals

Seven Long Evans rats, five females and two males, aged 6–15 months (Charles River Laboratories) were used in the study. All procedures were approved by the Institutional Animal Care and Use Committee and followed the National Institutes of Health guidelines. All rats were handled by laboratory staff for at least 3 d before testing to familiarize rats with the experimenters. Reward pellets (BioServ Dustless Precision Pellets) were placed in the home cage to forestall any neophobic reactions. Rats were food restricted to no <85% of their free-feeding weight to increase motivation for reward pellets. Rats were single housed (to avoid potential headcap damage) with a 12/12 light/dark cycle. All testing occurred during the light cycle.

### Housing design

**Figure 1.**
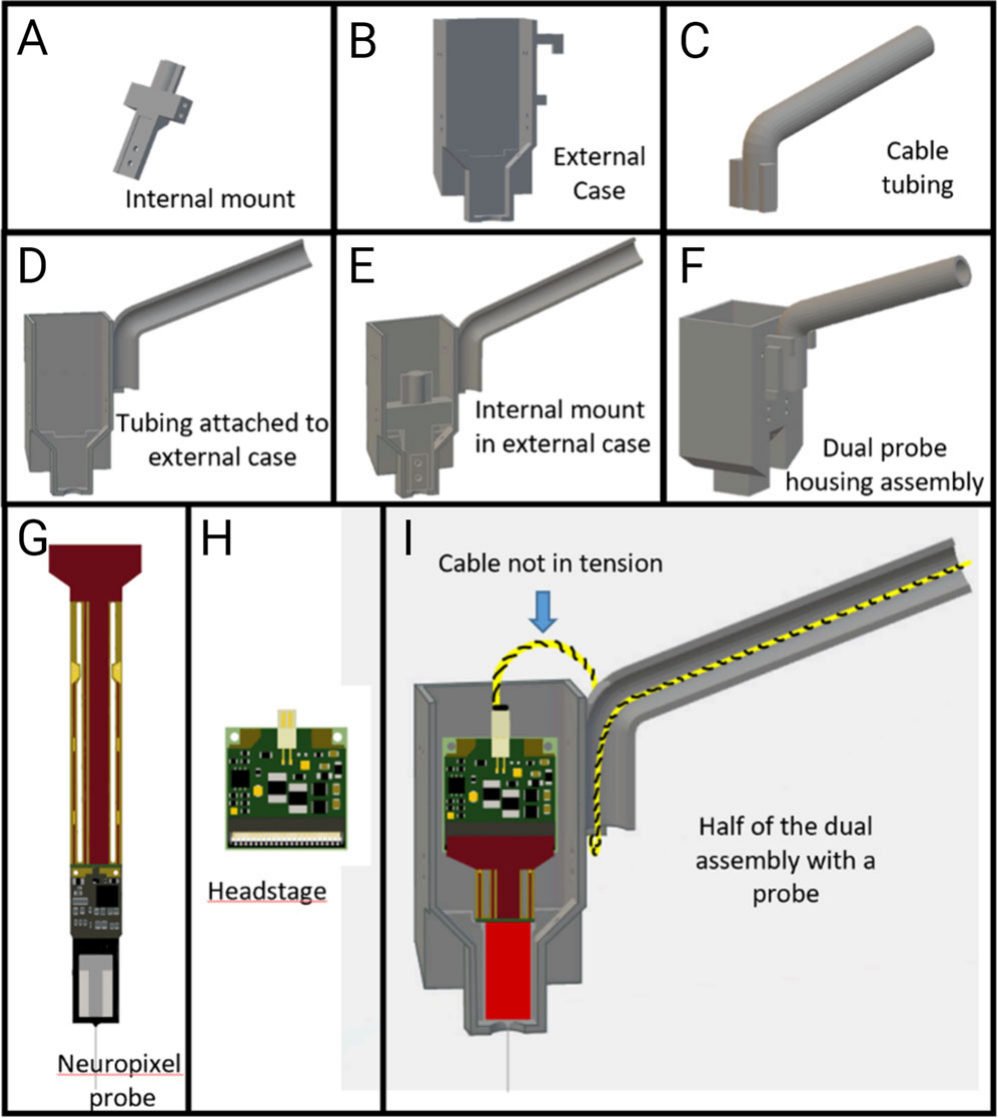
Schematic drawing of the Neuropixels housing design. Top row from left to right: internal mount (***A***), external case (***B***), and tubing (***C***). Middle row: assembly and relative position of the parts (***D–F***). Bottom row: a single probe and a headstage housed in half of the housing (***G–I***). Two halves glued together enable a dual site implant. The tubing, but not the recording cables inside, will be pulled during rat movement.

The primary goal of the housing design was to secure the probe for implantation and enable later explantation. As shown in Figure 1, our housing design is comprised of an internal mount, two halves of the external case (one for a single probe implant), and tubing to guide a recording cable while providing strain relief. The internal mount is for holding a Neuropixels 1.0 probe, which is then secured in the external case with four screws from the side. During implantation, the external case would be cemented to the rat skull, while no cement would touch the internal mount or the probe. This enables the explantation of the probe with the internal mount. The tubing holds and protects the recording cables from being pulled and the headstage Omnetics connector from being unplugged during rat movement, by retaining slack in the cable. In this design, the probe flex is connected with the headstage and both are tucked beside the internal mount. There is space between the internal mount and the back wall of the external housing. The probe flex would form a sideways “s” shape when in place (see below). The headstage is fixed in place by a thread going through the holes in the headstage and tied to the walls of the external case. There are two holes on each side of the walls just for that purpose. In this way, the headstage stays with the housing and the animal in between recordings. The advantages of leaving the headstage chronically connected within the housing are (1) each day's recording requires only the relatively easy step of plugging in the four-pin connector cable for each recording and (2) this design avoids repetitive clamping of the headstage to the probe flex. The connection between the ZIF connector in the headstage and the probe flex is sensitive to repetitive mechanical stresses and can be easily compromised/damaged over time. In our personal experience, a reused probe can require several clampings to be detected by the recording software. Hence, we developed this system to prevent damage to an unrepairable part. All the housing pieces can be 3D-printed using tough resin with a 50 µm printing resolution (https://formlabs.com/store/materials/tough-1500-resin/). The .STL files (including designs that should be suitable for Neuropixels 2.0 probes, though they have not been tested) are available at https://github.com/tne-lab/Neuropixel-methodology-manuscript/tree/main/STL files.

### Probe preparation

**Figure 2.**
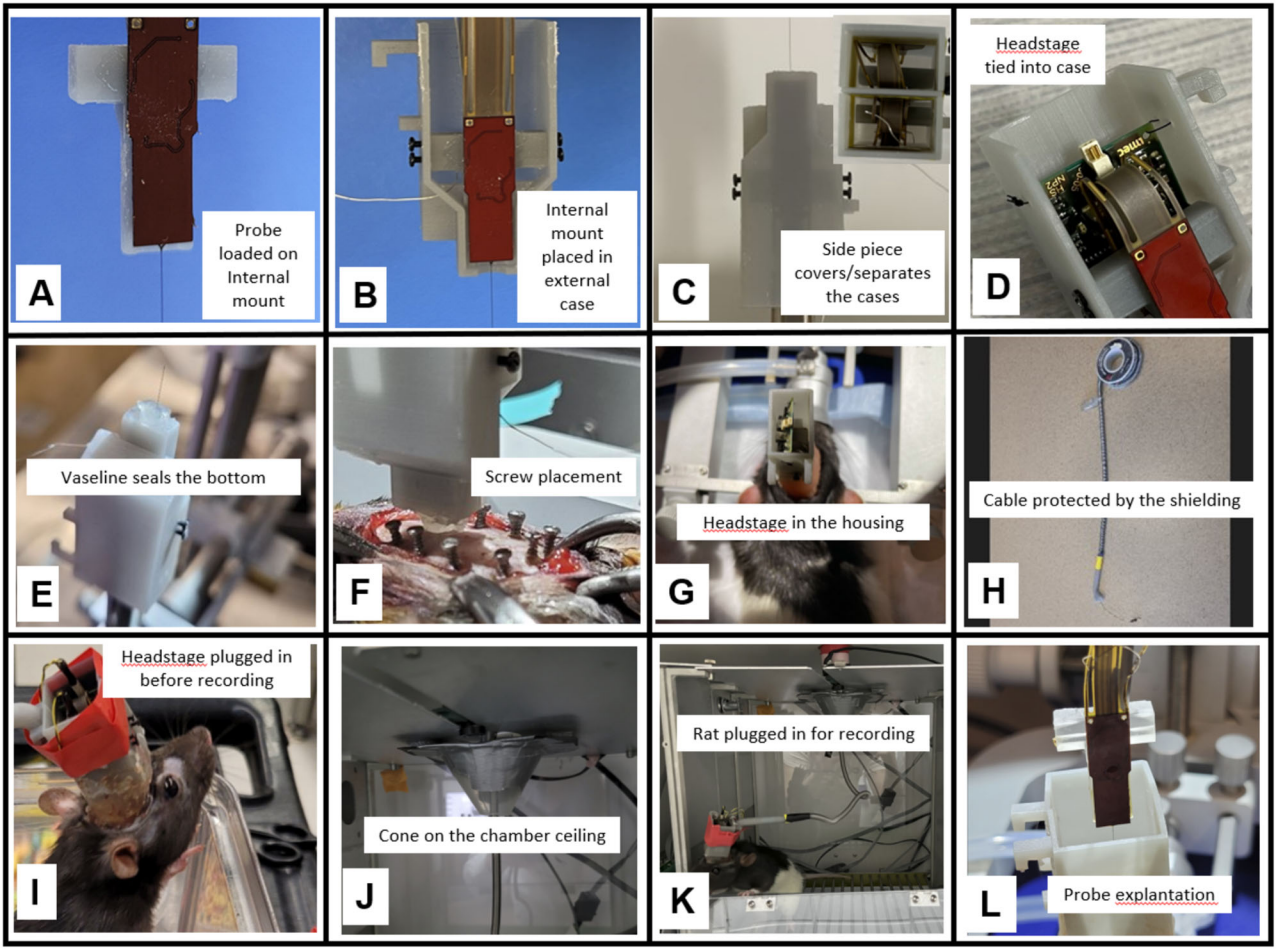
Neuropixels implantation and explantation. ***A***, Single Neuropixels probe secured in an internal mount. ***B***, The internal mount is secured within the housing by four screws. ***C***, Singular probe with lid on the housing (inset shows the lid separating two external cases). ***D***, A close-up view of the headstage and the probe flex when secured in the housing. The flex cable forms an “s” shape. ***E***, Singular probe housing sealed with Vaseline in the bottom, ready for implantation. ***F***, Probe lowering to target, showing anchor screw placement on the rat skull. ***G***, A headstage tucked in the housing when implanted. ***H***, Recording cables protected with metal spring. ***I***, A connected neuropixels probe rat. ***J***, Cone in the chamber ceiling to protect cables. ***K***, A connected rat in the chamber. ***L***, A probe that was just explanted.

One day prior to surgery, the probes were carefully taken out of the package and slid into the groove of the internal mounts (Fig. 2*A*) that were clamped to stereotaxic arms (Model 1766-AP Cannula Holder). To fully ensure that the probe was attached firmly, a small amount of Gorilla Glue (Gorilla Super Glue Gel XL) was applied to the area between the sides of the probe and the internal mount. It is critical to not allow glue on the sides of the internal mount to avoid inadvertently gluing the internal mount to the external case (which would make explant impossible). The offset of the probe shank from the edge of the internal mount can be finely adjusted based on the depth of the implant. Once the glue was dry (∼15 min), the internal mount was carefully placed into half of the external case by placing the case underneath the internal mount and pushing up the external case slowly (Fig. 2*B*). When the internal mount was completely placed in the external case, the bottom of the internal mount would be directly against the inner side of the bottom of the housing so the screw holes of the housing aligned with the screw holes of the internal mount. Each probe was secured inside half of the housing using four screws (0–80 thread, 1/16″ long, from McMaster) from the sides to stabilize the internal mount piece. The headstage would be later housed and secured at the end of the surgery between the back of the internal mount and the wall of the external case (Fig. 2*D*, also see *Surgery* session below). In a single probe implant, a flat side piece was then glued to the housing to close the side (Fig. 2*C*).

In the dual implant, the two halves of the housing were glued together to form a complete dual housing. In theory, the two shanks of the probes should be parallel to each other, but in practice small manufacturing irregularities cause displacement. After examining for misalignment by eye, we filed the edges of the housing or reloaded the internal mount to the housing to make sure the shanks were parallel.

Depending on the specific brain areas to be recorded, there may need to be an offset between the probes and their craniotomies. A flat side piece can be placed between the two halves of the housing to adjust the spacing between the two shanks (Fig. 2*C*). In our case, a side piece of 1 mm thickness was used between the two halves of the external case to ensure an appropriate spacing between the two shanks, and then the whole assembly was glued together. The precaution was taken not to apply the glue anywhere else, especially not on the internal mount. The entire assembly was then put aside to dry. On the surgery day, the probe assembly was placed under UV light for 30 min to sterilize. Fifteen minutes before the implant, fluorescent dye CM-Dil (Thermo Fisher Scientific) was applied to the shanks by applying the dye through a 27G needle with a syringe back and forth a few times along the tip of the shank. Finally, Vaseline was applied to the base of the shanks/bottom of the housing, and a cauterizer pen was briefly placed close to the jelly to melt it, to seal the probe exit (Fig. 2*E*). This was done to prevent fluid from coming into the housing in case the craniotomy was not sealed perfectly. We determined that, without this step, cerebrospinal fluid and/or tissue fluids would infiltrate into the housing. Over time, this humid environment damaged the sensitive probe electronics and caused both the probe and headstage to stop working.

Videos for the above probe preparation steps are available at https://github.com/tne-lab/Neuropixel-methodology-manuscript/videos.

### Surgery

The animal was mounted in a stereotaxic device following anesthesia using isoflurane (4–5% for the induction chamber and 0.5–3% for maintenance). The fur on the head was shaved off. An incision (1.5 cm in length) was made in the skin to expose the skull, which was then cleared of tissue and dried off. A drill bit (0.7 mm) was used to make 8 holes for support screws at the edges of the ridges surrounding the incision and one hole over the cerebellum for the ground screw (Fig. 2*F*). C&B Metabond was then applied to the base of support screws where they connected to the skull. Craniotomies for the probes were made using coordinates relative to bregma (for the hippocampal implant, −4.5AP, −2.5ML, −8.8DV, 19° angle; for PFC, +3.5AP, −0.7ML, −6.0DV; for the striatum, +2.0AP, −1.44ML, −7.0DV). A small amount of bone wax was applied in the craniotomy to seal it. The probes were inserted through the bone wax until they reached the target depth. Once the probes were lowered, the bottom of the housing was close to the skull, and the Vaseline applied to the base of the shanks earlier would encircle the shanks. The first layer of dental cement was then applied to the base of the housing. The ground wire was tied to the cerebellar screw. For dual-probe implants, ground wires were first wrapped together and then tied to the screw. A second layer of dental cement was applied to make the implant secure and complete the headcap. The headstage was connected to the probe flex via the ZIF connector and tucked gently into the housing. It was then tied with suture material to the four holes to the sides of the housing (Fig. 2*D,G*). The headstage stayed attached to the probe during the entire experiment, such that for each testing day, recording only required connecting the headstage to the recording cable using the four-pin Omnetics connector. This avoids repetitive clamping and thus facilitates long-term recordings. The four-pin connector was protected with the 3D-printed tube system (Fig. 1*I*) such that it would not be pulled during the rat movement (no tension). Additionally, the headstage could be easily replaced if needed by cutting the suture that tied it to the wall of the external case. Lastly, a piece of paper tape was used to cover the top of the housing. The animal was then allowed to recover for a week before behavioral training resumed.

### Electrophysiological recording

Prior to the recording, rats were head-touched while rewarding them with sugar cereal for 3–5 min each time for 5–10 times across several days. This was to train them to keep still for the connection between the recording cable and headstage. By shaping them to be calm for connection, rats were unrestrained; thus, any possible stress was minimized. Prior to the recording, the portion of the interface cable that would be below the chamber ceiling was shielded with a metal spring sheath from Plastics 1 (Fig. 2*H*). One end of the metal spring was attached with electrical tape to the tubing that was 3D printed (Fig. 1*C*). The other end of the spring was connected to a commutator that was fixed on the ceiling of the chamber. During movement, all forces were transmitted to the spring sheath and to the attached tubing, which then would turn the commutator. The interface cable was loose inside of the metal spring and was protected against being unplugged by tensile force and was protected against bites/chewing by the spring sheath. A cone-shaped plastic cover (Fig. 2*J*) was used to cover the cable around the commutator to avoid any possible manipulation or chewing by the rats. This design prevents the recording cables from being pulled or placed under tension by rat movement (Fig. 2*K*). The setup allowed for recordings that covered the entire task session (up to 1 h, though a longer period has not been tested). Recordings were conducted in a Coulbourn Instruments behavioral chamber up to 1 month postimplantation.

### Probe explant

Probe explant was performed in a stereotaxic device with the animal under anesthesia (isoflurane, 2–5%). The screws holding the probe holder in place in the housing were removed and the threads holding the headstage in place were cut. The headstages were then removed. With the animal's head stabilized by the ear bars, a cannula holder (Kopf, Model 1766-AP) was used to clamp the internal mount with the probe, and a stereotaxic arm was used to pull it straight up and out of the housing (Fig. 2*L*). The probe was then soaked in 1% Tergazyme solution (catalog #16-000-115, Fisher Scientific) overnight and distilled water for 2 h to clean off any tissue.

### Histology

Immediately after the probes were explanted, the rat was given an intraperitoneal injection of a pentobarbiturate (Euthasol). Once deep anesthesia was ensured, rats were transcardially perfused and brains were fixed with 4% PFA for 48 h. Brains were placed in 30% sucrose for at least 48 h and then sliced into 40 µm coronal sections. The sections were first imaged for fluorescence using a Keyence microscope and then Nissl stained using cresyl violet to confirm probe placements.

### Data analyses

MATLAB v2021a was used in data analyses. The Chronux package and the analysis tools from Open Ephys GitHub were used to read the data and calculate power and coherence. Raw local field potentials (LFP) data were re-referenced by subtracting from each channel the average of five neighboring channels. Spike sorting was conducted using Kilosort3 and Phy2. Well-isolated single units were identified by Kilosort3 and verified by Phy2 visualization. All the scripts are available at https://github.com/tne-lab/Neuropixel-methodology-manuscript/code. We performed analyses to verify the yield of single units, represented as unit counts across days and implants. To demonstrate consistency in the gain and noise characteristics of explanted and reused probes, we calculated the root mean square (RMS) of the signal to estimate noise levels across multiple days for new, reused, and twice reused probes. Without knowing the ground truth of the noise level from an implanted probe, the RMS is one way to estimate that noise, because increases in non-physiologic noise levels would be reflected in the RMS. To further characterize performance in frequency domain analyses, we used the FOOOF method (Donoghue et al., 2020), as implemented in the FieldTrip toolbox (https://www.fieldtriptoolbox.org/example/fooof/) to calculate the 1/f noise and 1/f removed θ-band power for all 384 channels. We plotted the number of channels that had above zero “clean θ” power across probes with different implantation conditions. To illustrate the potential value of multi-probe implants, we performed an example calculation of coherence between cortical and striatal regions during the set shift task (see below). Raw coherence was calculated using intact data from all behavioral epochs. To account for bias in that calculation, shuffled coherence was also calculated after the data from all epochs (from both regions) were randomly shifted in time to destroy consistent phase relationships. Corrected coherence was then calculated as the shuffled subtracted from the raw coherence. This was performed to more accurately represent connectivity between the two brain regions.

### Behavioral tasks

#### Paired associate learning task (PAL)

**Figure 3.**
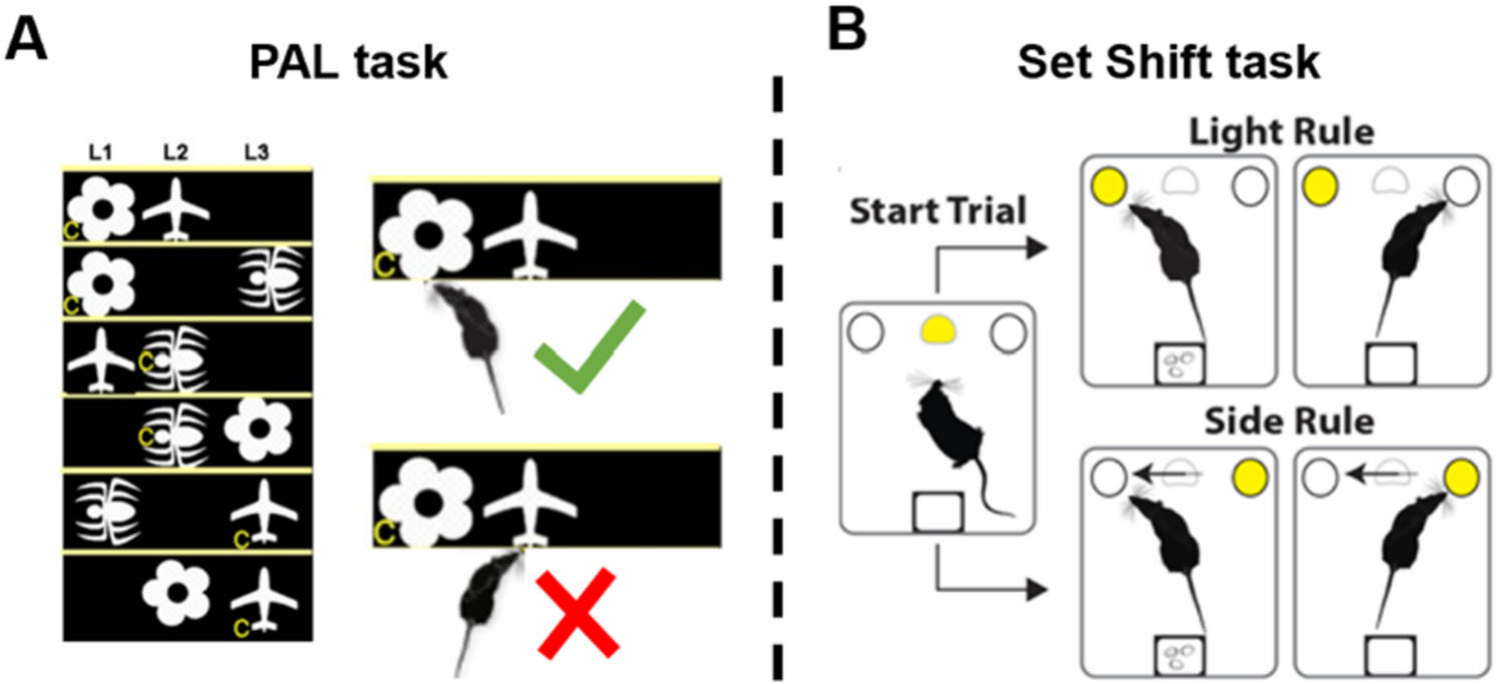
Paired associate learning task and set shift task. In the PAL task (left), rats were shown a pair of images on a monitor in two of three locations. Touching one of the images led to a sucrose pellet reward and touching the other led to a 10 s time-out. The correct image was determined by the intersection of image identity and location, e.g., the flower is correct only in the left spatial location and the airplane only in the rightmost. In the set shift task, rats needed to learn to nosepoke into an illuminated port (Light rule) or into a port on one specific side of the chamber (Side rule) to earn a reward. The rule shifts without warning and must be discovered through trial and error.

The PAL task (Fig. 3*A*) is a spatial memory task as described in (Talpos et al., 2009). Briefly, rats (*n* = 3) were first trained to nosepoke or pawtouch a touchscreen that was divided into three rectangles using a metal mask. Each rectangle represented the correct location for one image. During the task, two images were shown on the screen—one in the correct location and one in the incorrect location, with the third location blank. Nosepoking the correct location resulted in the delivery of one sugar pellet, while choosing an incorrect location resulted in a 10 s time-out. Image pairs were presented in a pseudorandom order, and the task was finished once the rat got 100 image pairs correct or 1 h had elapsed.

#### Set shift

We conducted a set-shifting task as described previously (Darrah et al., 2008; Floresco et al., 2008; de Oliveira et al., 2021). Briefly, rats (*n* = 4) were trained to switch between two rules, a “light rule” where they must nosepoke a port that was illuminated, and a “side rule” where they must nosepoke either the port in the front or the back of the operant chamber no matter which port was illuminated (Fig. 3*B*). A correct nosepoke earned them one sugar pellet. The rat was required to respond correctly to five trials of a rule in a row to proceed to the next rule and an incorrect nosepoke reset to the beginning of the rule. There was no cue to indicate when the task switched from one rule to the next, so the rat was required to infer the rule based on the recent history of (non)reward. The order of the rules was switched pseudorandomly between days so the rat could not memorize the order of the task. The task was completed once the rat completed 8 rule shifts or 90 min had elapsed.

## Results

**Figure 4.**
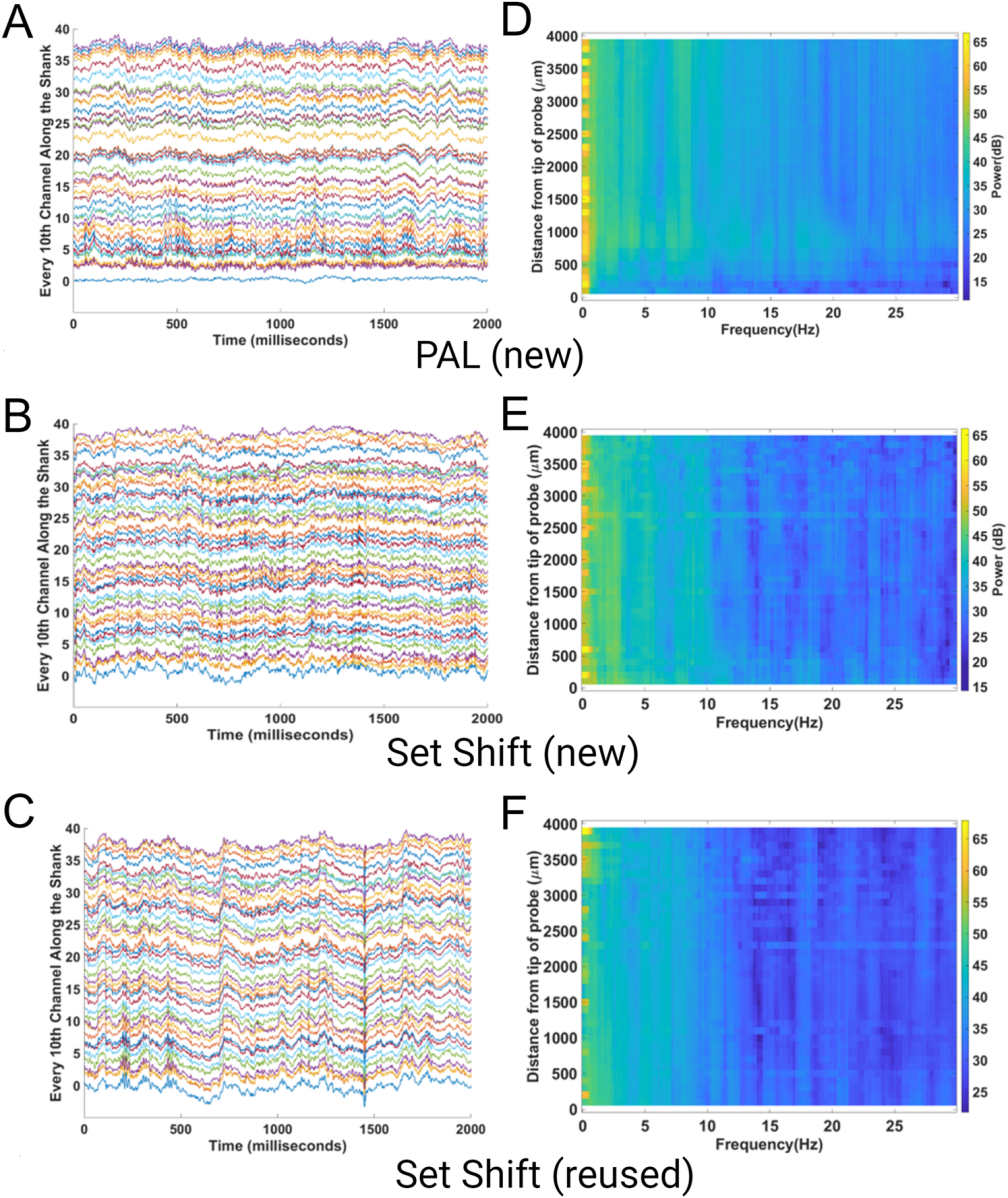
LFP recordings from the PAL and set shift tasks. Shown are representative raw traces of every 10th channel along the first bank of the shank (***A***,***B***) and power spectra of the signals along the first bank (***D***,***E***). ***C***,***F*** Raw traces and power from a reused probe.

**Figure 5.**
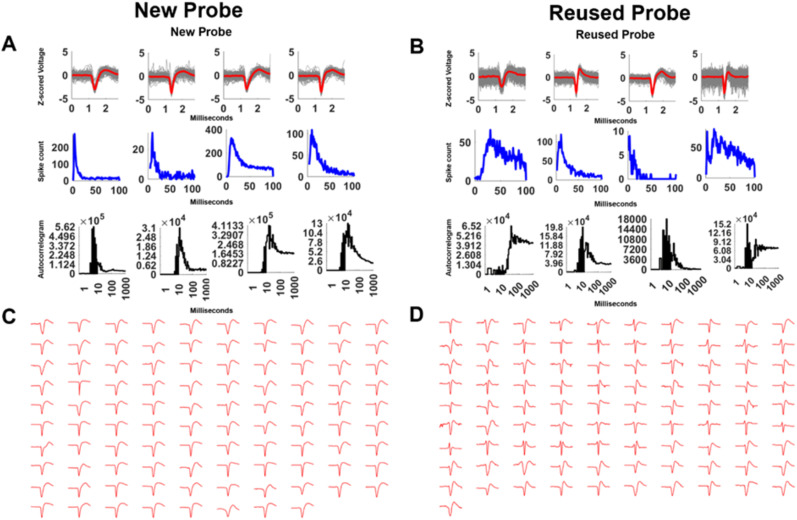
Spike recordings from new and reused Neuropixels 1.0 probes. Shown are representative putative single units recorded from a new probe and a reused probe. ***A***,***B*** Raw traces, inter-spike intervals, and autocorrelation of four representative putative units. ***C,D*** Each shows all the “good” units from a single recording.

**Figure 6.**
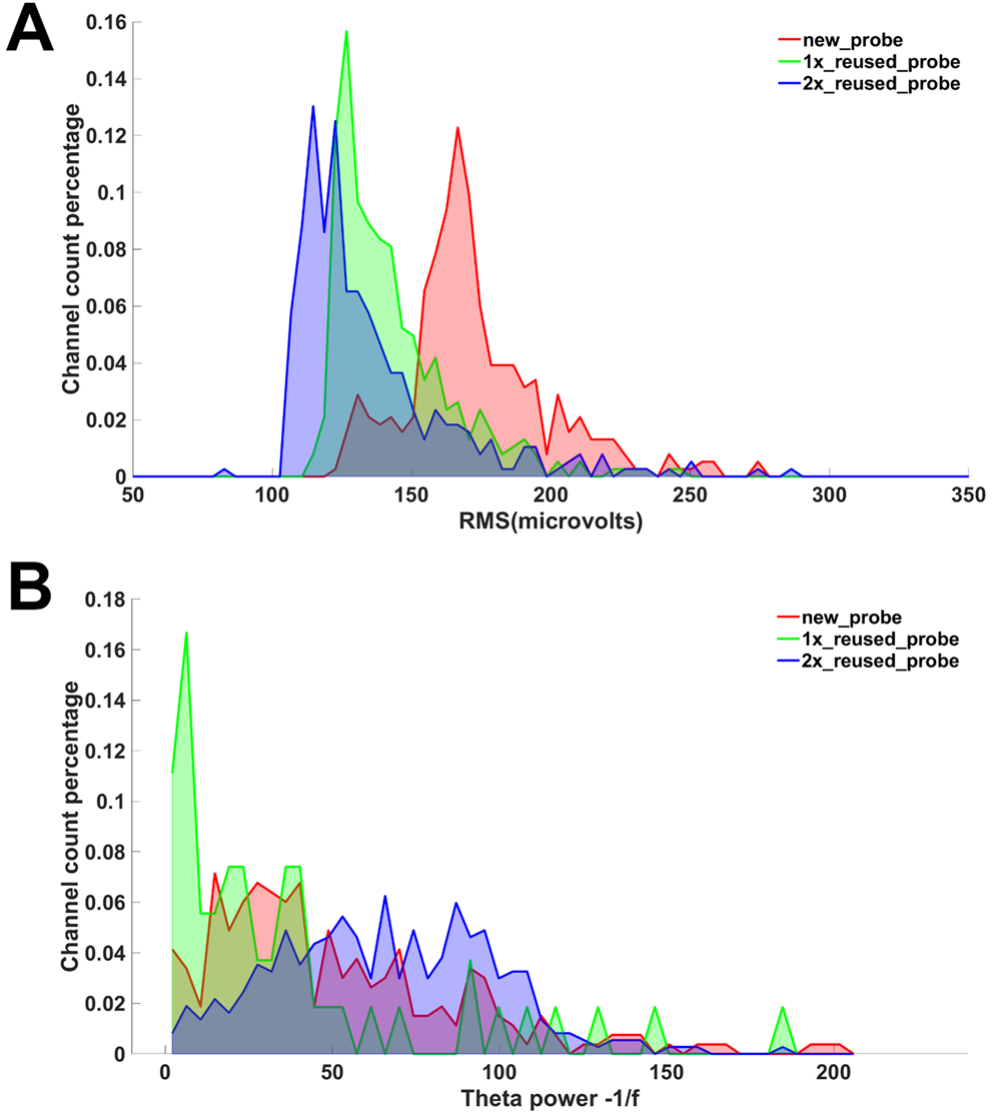
Signal noise and θ-band power in representative new, reused, and twice reused Neuropixels 1.0 probes. ***A***, Distribution of RMS noise for all 384 channels, for new and reused probes. ***B***, Distribution of θ power over 1/f noise for all channels that had positive power values (suggesting there were clean physiological θ band oscillations), for new (144 channels), reused (333 channels), and twice reused (158 channels) probes. Data were collected from the probes implanted in the prelimbic cortex in three rats. For each probe condition, over 75 min worth of data across multiple sessions was used.

**Figure 7.**
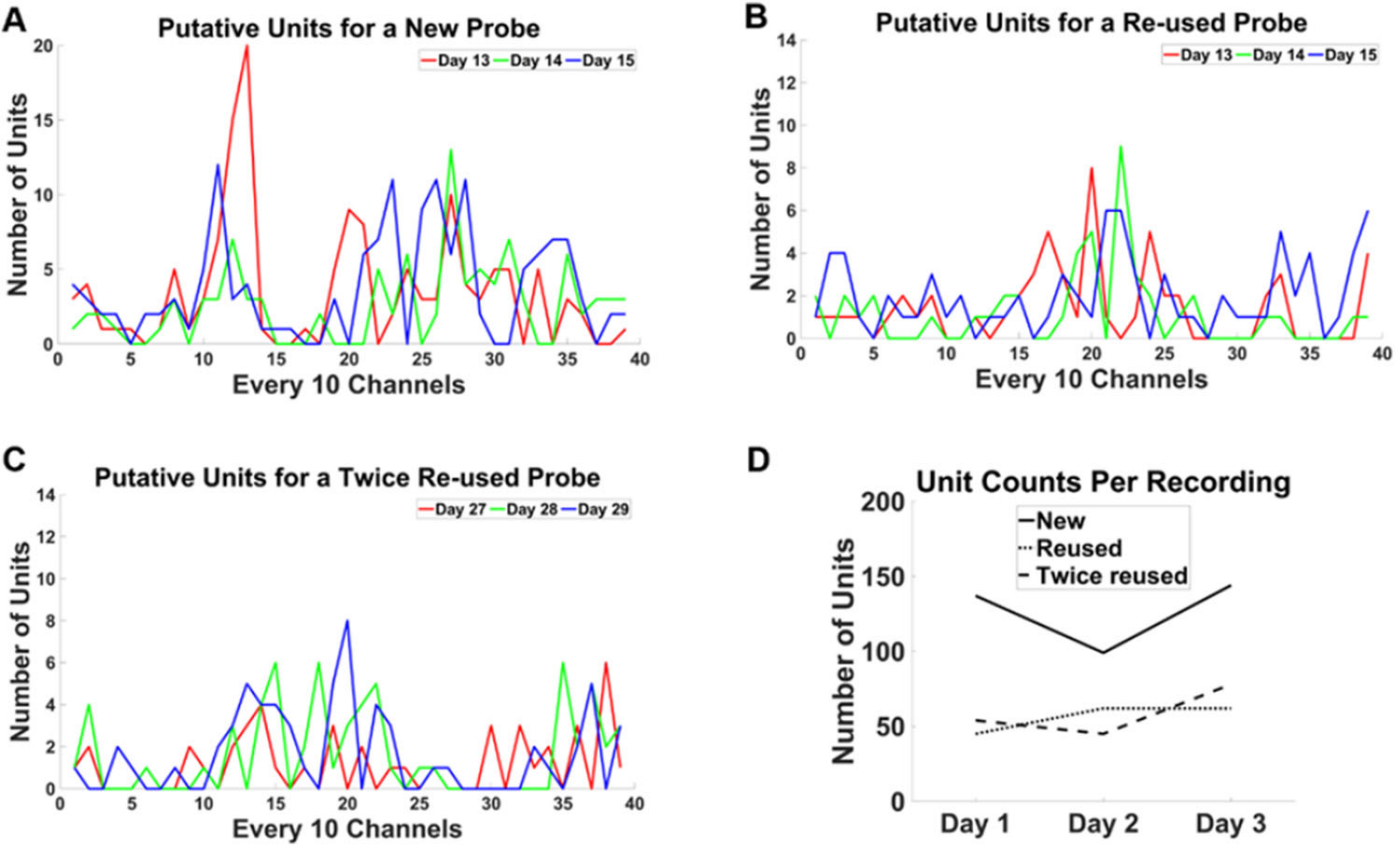
Unit counts from 3 consecutive days with new and reused Neuropixels 1.0 probes. Shown are unit counts of every 10 channels along the shank for representative new and reused probes. ***A***, Recordings from days 13, 14, and 15 postimplantation of a new probe. ***B***, Recordings from days 13, 14, and 15 postimplantation of a reused probe. ***C***, Recordings from days 27, 28, and 29 postimplantation of a twice reused probe. ***D***, Summary of ***A–C***, showing total unit counts along the probe for the 3 consecutive days shown in each panel. Although there was a clear drop from these new to reused probes, there was no evident loss across days.

Over 40 sessions of recordings were performed for this study. Recordings in the operant chambers showed clean LFP signals. Figure 4 shows example raw data and power spectra from every 10th channel of the 384 channels recorded during both the PAL and set shift tasks. Each recording session yielded 87.3 ± 10.1 (mean ± SEM) putative single units; for representative putative single units, see Figure 5. Two probes were reused once, and one other probe was reused twice. The average good unit yield of new probes was 91.9 ± 12.8 (mean ± SEM) units/probe, the number of units from once reused probes was 91.5 ± 16.0 (mean ± SEM) units/probe, and the number for the twice reused probe was 50.6 ± 5.2 (mean ± SEM) units/probe. The noise characteristics of the new and reused probes were examined by estimating the noise levels of the signal across multiple days. The RMS levels of the reused and twice reused probes were comparable and not higher than that of a new probe (Fig. 6). Similarly, the LFP θ (4–8 Hz) oscillation power above the 1/f background was not lower than that of a new probe (Fig. 6). The spike yield seemed stable during the implantation. Across multiple days, the number of units recorded at each site along the shank was qualitatively similar and did not decrease over time (Fig. 7). The number of recorded units was also consistent within a location on the probe (Fig. 7).

**Figure 8.**
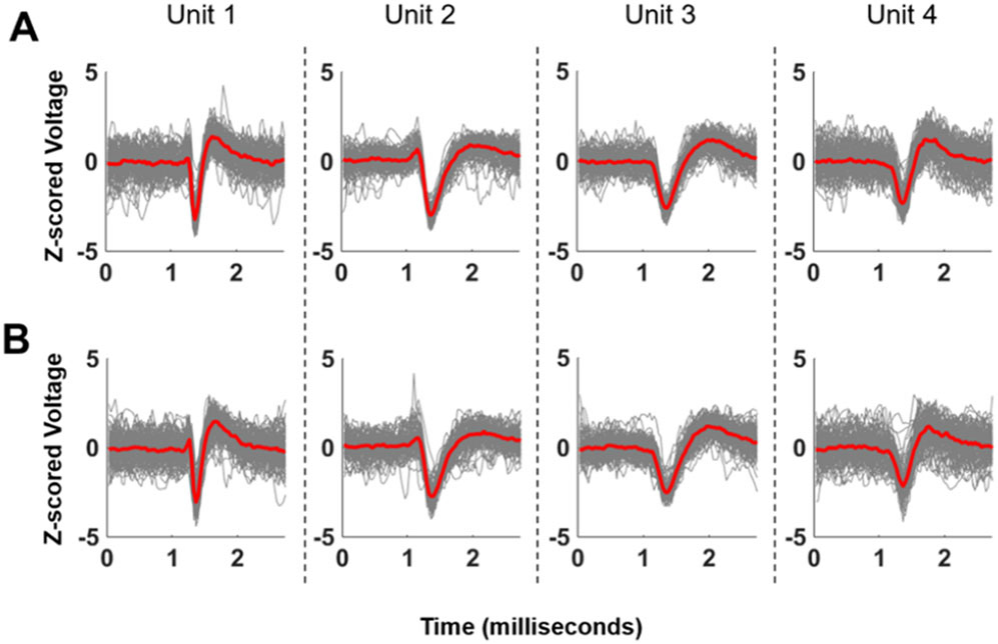
Waveforms of representative units are stable within a recording session. Panel ***A*** shows the first 100 spikes of selected units from one session. Panel ***B*** shows the last 100 spikes of the same units from the same session. The waveforms remain similar over the recording (*R* = 0.9929).

**Figure 9.**
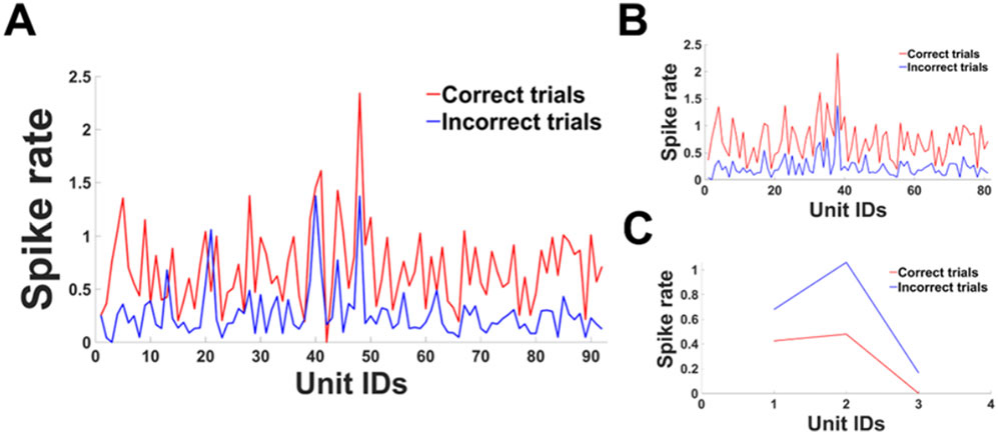
Capture of behaviorally relevant units with a reused probe during the set shift task. ***A***, Spike rates of all units during correct versus incorrect trials. ***B***, All units that had at least 1.5 times higher firing rates during correct versus incorrect trials. ***C***, All units that had at least 1.5 times higher firing rates during incorrect versus correct trials. Data were from the prefrontal cortex probe in a representative set shift session of one rat. Spike rates were averaged over the time period between cue onset and response.

**Figure 10.**
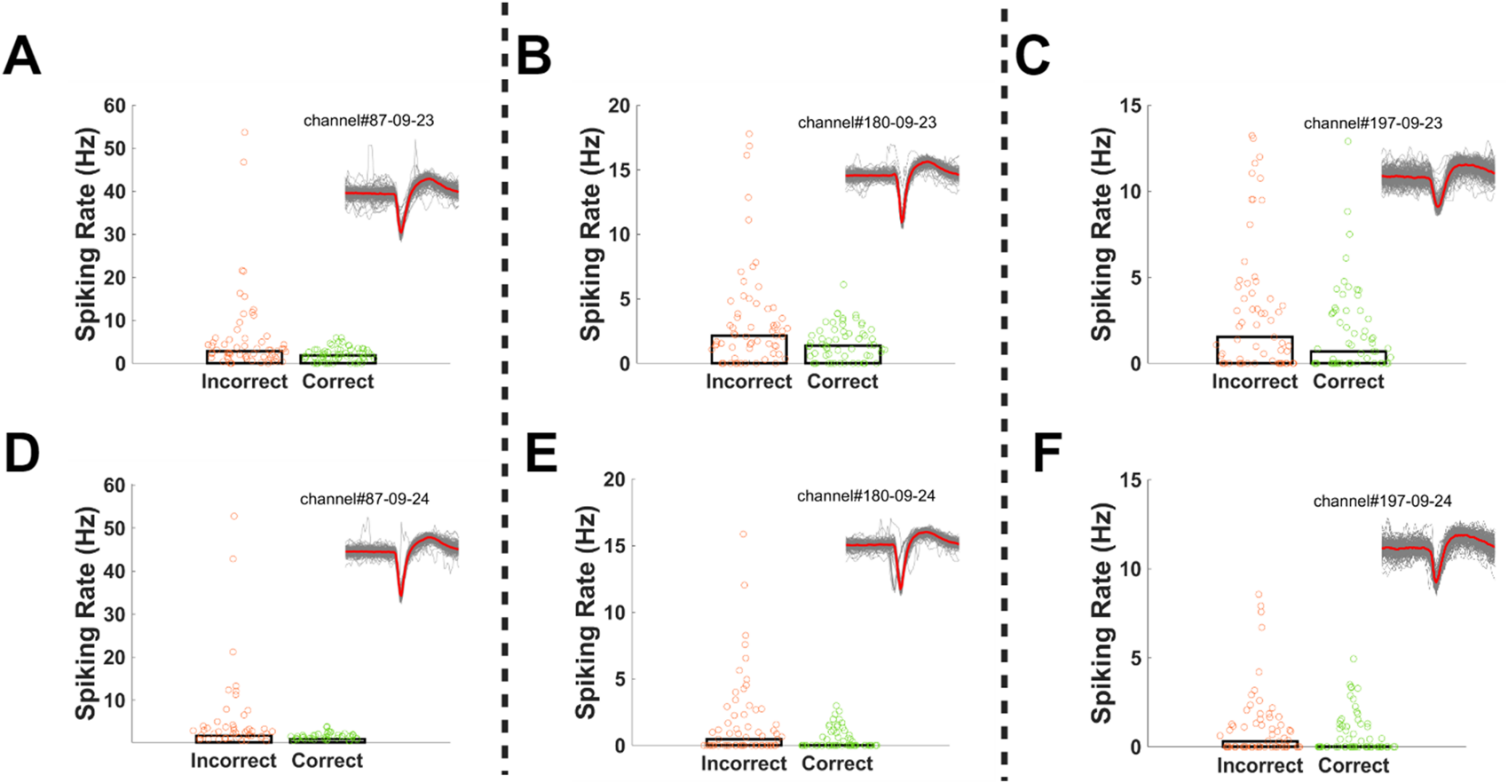
Representative units that had similar spiking patterns across days. Units A and D were located near the same channel of the probe and had similar spiking modulation during the incorrect and correct trials. The same is true for putative for units B and E and C and F. Waveforms were highly correlated across days (*R* = 0.9558). While not conclusive, the concordance of the waveform, anatomic location, and behavioral modulation suggests these are the same cells being tracked across days.

The housing tethers the probe to the skull via the screws and sutures, but the brain can move relative to the probe. As such, it is possible that movement during a recording session might disrupt single-unit capture beyond the level that can be compensated for by Kilosort's drift correction methods. This did not appear to occur. The waveforms of well-isolated units were stable across the recording session (*R* = 0.9929, Fig. 8). The captured units were behaviorally relevant. For instance, we identified units that differentially modulated their firing rate during trials where rats made correct versus incorrect responses, during a set shift session (Fig. 9). Further, some of these units were potentially traceable across days, in that we could identify sorted units in the same location on the probe with similar waveforms (*R* = 0.9558 between average waveforms) and behavioral modulation across days (Fig. 10).

**Figure 11.**
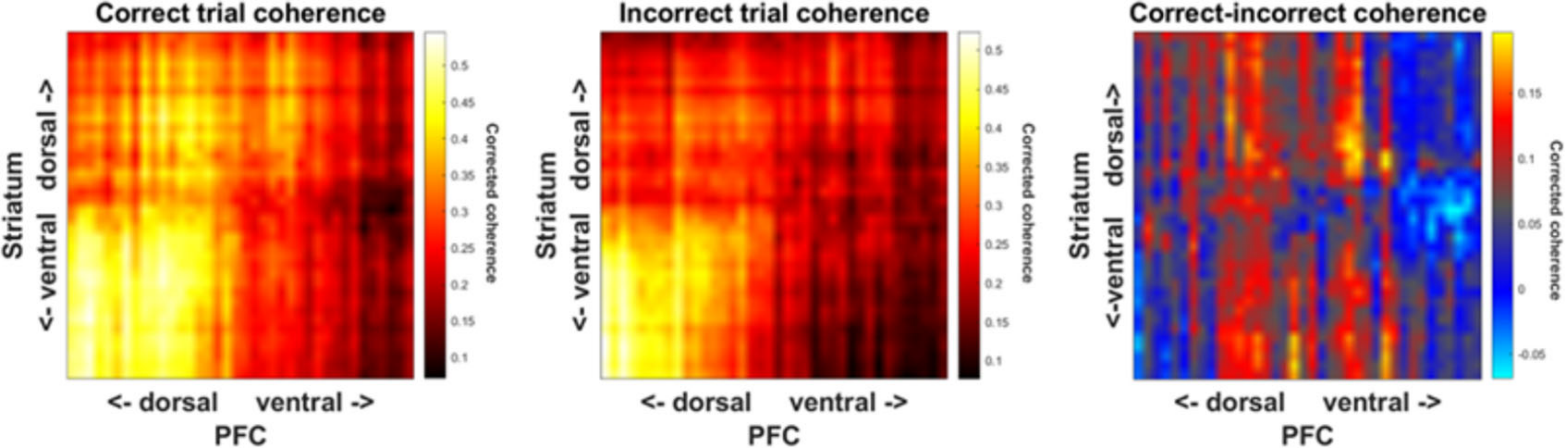
Coherence between the PFC and the striatum during the set shift task. ***A***, θ coherence between the PFC and the striatum in the set shift task during Correct trials. ***B***, θ coherence between the PFC and the striatum in the set shift task during Incorrect trials. ***C***, Difference in θ coherence between the PFC and the striatum in correct versus incorrect trials, showing interactions between dorsal and ventral PFC and striatum. Plotted are example data from one task session (∼45 min of recording). The 384 channels were grouped into 38 clusters with 10 channels as one cluster. A sliding window size of 0.5 s and a step of 0.1 s were used to calculate coherence. The corrected coherence was calculated from raw coherence subtracted by coherence from shuffled raw data.

A major value of the reported approach is the potential for measuring connectivity and communication across regions during complex behaviors. Breakdowns in circuit communication are alleged to be the primary mechanism of cognitive and emotional disorders. As a demonstration of the potential value of multisite Neuropixels measurements, we calculated the coherence between the PFC and the striatum during correct versus incorrect trials. We saw differences in θ (5–8 Hz) coherence between these trial types (Fig. 11), consistent with a reported role for θ-band synchrony in cognitive control and set-shifting (Cavanagh et al., 2011; Provenza et al., 2019; Widge et al., 2019). In general, coherence was higher during correct trials, consistent with the thesis that this task requires strong cortico-striatal interactions. While extremely preliminary, i.e., not taking into account learning processes or specific events during set shift trials, this result highlights the potential utility of this recording approach.

## Discussion

We report an improved protocol and housing design that reliably allows chronic, multi-probe Neuropixels recordings in free-moving rats in operant chambers. The two main improvements are a design to retain the headstage chronically connected to the probe (avoiding manipulation of a fragile component) and a strain relief and shielding to prevent cable unplugging and damage from a free-moving animal investigating the cable. Like prior protocols, this protocol allows reuse of the Neuropixels 1.0 probes after being chronically implanted in a rat. The explanted probes have stable performance in both LFP and spike recordings, with good unit yields and signal-to-noise ratio even on a second reuse. There is clearly a decrease in performance from a fresh probe, but likely not sufficient to prevent most investigations.

The present protocol has the headstages held in the upper space of the housing so that the bottom of the housing can be small enough for multiple probe implantation. Chronically connecting the headstage to the probe is desirable because it is difficult to clamp the probe flex to the headstage with a free-moving rat (van Daal et al., 2021), and repetitively clamping the ZIF connector on the headstage could cause damage to the connection. We believe that this is one of the major differences/improvements in the present design compared with the design reported by (Luo et al., 2020). Other prior designs had the headstages connected with the probe, but they were either unsuitable for multiple probe implantation (Juavinett et al., 2019) or did not adequately address the cabling issues with a free-moving animal in a relatively small operant chamber (van Daal et al., 2021).

The current cabling system uses metal shielding and a spring to protect the recording cables. The cables were not in tension because only the metal shield was attached to the housing via the 3D-printed tube, and this shield is shorter than the recording cables. All movement affected the metal shield and not the cables inside. The recording cables above the chamber would be wrapped at the ceiling of the chamber. Given the small diameter of the cables, wrapping around the commutator and having a plastic cone holding the cables worked well and prevented rats from getting to them. This setup does not use two sets of cables above and underneath a commutator. Instead, it uses one set of cables attached to the commutator and allows the cables to wrap around it. The recently developed ONIX system (https://open-ephys.org/next-gen-acquisition-system#features) does have a torque-free commutator and real-time 3D orientation tracking in the headstage, which should work well in an arena such as an open field. However, the ONIX system may not resolve the problem of cable tension in operant chambers, particularly with larger rodents (rats). Thus, our protocol provides an intermediate solution while the ONIX system is being further optimized in that regard.

The dual housing design consists of two single housings that can be used combined or independently if needed, making it flexible in targeting two brain sites at various distances apart. Two single-housing external cases can be directly glued together with a spacer piece (of varying thickness) to adjust the distance between probes. This allows customization to reach any two specific targets in the brain. The two external cases can also be used independently of each other, each with its own side piece. This can be useful when the two brain targets are far apart or one or two probes need to be lowered down with an angle. In this case, the hooks for the cable tubing can simply be moved to one of the external cases.

Limitations of this protocol include its slightly taller housing compared to prior designs, which required containing the headstage chronically. This size, however, did not affect behavior as our implanted rats seemed to perform normally after implantation. Additionally, the cabling system is still not the best solution as it does not really utilize the “functionality” of a commutator. The cables will wrap around a commutator as rats move about in an operant chamber. The standard recording interface cables are flexible, 5 m long and 410 × 820 µm thin. Wrapping around a commutator protected by a plastic cone works well in most cases. However, this should still be improved in future designs. Finally, although we did not observe substantial 60 Hz noise in our preparation, this might be because we used grounded metal chamber floors. Other arenas, particularly with insulating floors, might require further mitigating measures. These might include shielding the probes such as by painting the case with conductive silver paint or wrapping thin foil around the case and grounding it through a small wire.

In summary, this study reports an improved protocol for chronic Neuropixels recordings in operant chambers with small rodents. The protocol has a robust cabling system that enables stable recordings and prevents chewing and manipulation by animals. It keeps the headstages connected with the rats and avoids repetitively connecting to the Neuropixels probes for each recording. The protocol also allows implantations with multiple probes and reuse of the Neuropixels probes after explantation. We demonstrate here results with the more widely used Neuropixels 1.0 model and have provided template files to extend this to Neuropixels 2.0 work. Overall, this method should facilitate the usage of chronic Neuropixels recordings in future research.

## Synthesis

Reviewing Editor: Jibran Khokhar, Western University Department of Anatomy and Cell Biology Decisions are customarily a result of the Reviewing Editor and the peer reviewers coming together and discussing their recommendations until a consensus is reached. When revisions are invited, a fact-based synthesis statement explaining their decision and outlining what is needed to prepare a revision will be listed below. The following reviewer(s) agreed to reveal their identity: Thomas Luo, Lucas Sjulson.

As is clear from both the reviewers, including more data would help to show that this method does work and solves problems previously faced, and is superior to other approaches. Addressing the comments from the reviewers will help to make this paper more impactful and clarify the benefits of this approach.

## Author Response

Synthesis of Reviews:

Significance Statement Comments for Author (Required):

N/A

Comments on the Visual Abstract for Author (Required):

N/A

Synthesis Statement for Author (Required):

Both reviewers felt that the authors did a great job of responding to comments. One of the reviewers raised the following minor points that will help to improve the manuscript:

To increase clarity, the authors can consider labeling the panels In Figure 1 with letters and reference the panels using these letters in the legend to Figure 1.

Done, thanks.

In the Figure 4 legend, by "dPAL," did the authors mean "PAL?"

Yes. This is now corrected. Thanks.

Can the authors provide more details regarding the following: "To demonstrate consistency in the gain and noise characteristics of explanted and re-used probes, we calculated the root mean square (RMS) of the signal noise across multiple days for new, reused and twice reused probes." What do the authors mean by "signal noise?" Are these recordings from the probe in saline?

The root mean square is the square root of the arithmetic mean of the squares of the voltage values. Without knowing the ground truth of the noise level from an implanted probe, the RMS is one way to estimate signal noise, in a way that any high signal noise would be reflected in the RMS. The fact that we did not see high RMS values in re-used probes indicates the reusability of the probes. Recording in saline would be a way to estimate the noise level, but we did not systematically test each probe in saline prior to implantation. We can do that moving forward. Please also see our response to the last point in this document. The text has been revised as following:

"To demonstrate consistency in the gain and noise characteristics of explanted and re-used probes, we calculated the root mean square (RMS) of the signal to estimate noise levels across multiple days for new, reused and twice reused probes. The RMS is the square root of the arithmetic mean of the squares of the voltage values. Without knowing the ground truth of the noise level from an implanted probe, the RMS is one way to estimate that noise, because increases in non-physiologic noise levels would be reflected in the RMS.".

The authors may wish to move or paraphrase these two sentences in their Methods to the Introduction to further motivate the use of the Omnetics connector rather than the ZIF connection: "In our personal experience, a re-used probe required several clampings to be detected by the recording software. Hence, we developed this system to prevent damage of an unrepairable part."

Thanks. It is revised in the Introduction as follows:

"Some of the existing designs also require re-connecting the probe to the headstage each day (Luo et al., 2020). This is challenging, as it requires precise manipulation of a very small part while the animals are moving. In our experience, a re-used probe could require several clampings to be detected by the recording software. Further, repetitive clamping with the zero-insertion-force (ZIF) connector in the Neuropixels headstage often leads to damaged connections due to repeated mechanical actuations of a small, fragile part. "

Can the authors provide pictures or schematics to graphically illustrate this part of the Methods: "Depending on the specific brain areas to be recorded, there may need to be an offset between the probes and their craniotomies. A flat side piece can be placed between the two halves of the housing for adjusting the spacing between the two shanks. In our case, a side piece of 1mm thickness was used in between the two halves of the external case to ensure an appropriate spacing between the two shanks, then the whole assembly was glued together."

The picture is now included as an inset in Figure 2C.

In line 292, if the authors can modify this sentence in the results to include the metric "unit/electrode," that would be helpful: "Each recording session yielded 87.3{plus minus}10.1 (mean{plus minus}SEM) putative single units; for representative putative single units"

By "single unit," do the authors mean the "good" units identified by Kilosort3?

Yes, "single unit" means the good units identified by Kilosort3 and verified manually. The text has been revised in the Data Analyses section.

"Well isolated single units were identified by Kilosort3 and verified by Phy2 visualization."

The metric "units/probe" is now included in the text.

Line 295-296, "The noise characteristics of the new and reused probes were examined by calculating the noise floor." Is this done saline or in vivo? If done in vivo, how are the authors removing the "signal" from neural spiking activity from their calculation of the noise floor?

We used both the root mean square (RMS) and the aperiodic portion of the existing raw signals calculated by the fooof algorithm to estimate the noise levels. Without knowing the ground truth of the noise level, these are the best approaches that are available for noise estimation. The text has been revised as the following:

"To demonstrate consistency in the gain and noise characteristics of explanted and re-used probes, we calculated the root mean square (RMS) of the signal to estimate noise levels across multiple days for new, reused and twice reused probes. The RMS is the square root of the arithmetic mean of the squares of the voltage values. Without knowing the ground truth of the noise level from an implanted probe, the RMS is one way to estimate that noise, because increases in non-physiologic noise levels would be reflected in the RMS.". To further characterize performance in frequency domain analyses, we used the FOOOF method (Donoghue et al., 2020), as implemented in the fieldtrip toolbox(https://www.fieldtriptoolbox.org/example/fooof/) to calculate the 1/f noise and 1/f removed theta band power for all 384 channels.

"The noise characteristics of the new and re-used probes were examined by estimating the noise levels of the signal across multiple days. The RMS levels of the reused and twice reused probes were comparable and not higher than that of a new probe".
